# Professional Personalities

**Published:** 1858-03

**Authors:** 


### PROFESSIONAL PERSONALITIES.

Many good operators underrate the importance of office etiquette, and in failing to improve themselves in this particular, lose a most valuable means of accomplishing much good to their applicants for dental services, and to themselves, the best reward of success. We are essentially a fast people, and dentists are particularly rapid in everything. American dental reputation is the most current in all the civilized countries of the world. As a proof of this we see our brethren most popular in all the great cities of Europe, and South America. Yet at home we occasionally neglect those little courtesies which, in the minds of many, constitute the power of man to draw others to his purpose.

Talent and skill, however available, are not the only requisites of usefulness, not the only means of placing professional attainments in their proper position before a public. If it were so, how could Homeopathy or Hydropathy, or Thompsonianism draw so successfully at times upon popular credulity and patronage. Standing in Fourth Street of this City, you are almost induced to imagine that you see some Esculapius rolling along in his jaunty vehicle, when it is simply a successful pellicle embedded in cushions, stuffed by the bank paper of his simple adherents, who have been charmed with the gentleman's address and the simplified description of the "*Tyserinctum*," and its modified conditions under the influence of morbid action. Or you are sometimes induced to believe, from some beauty, that drowning in the hands of Dr. Hydrantale, is a reliable cure for your especial troubles; and a delightful old lady neighbor, induces you to believe that vaporization, as applied by Dr. Gasopen, is the natural means of purifying the body and soul of all ills. Still educated dentists mostly manage to keep from these delusions, and we, therefore, drop the subject in denying to these masters any other knowledge than that of personal fascination; for it is useless and worse, to reason against such matters as have not a shade of science or philosophy, that can be so called, to support them.

Although our art deals altogether in positive demonstrations, and in tangible facts, yet there is a great tact required in inducing subjects to submit to these not always pleasant, illustrations. We have to do that which no other profession can claim to do ; that is, by using certain means to produce certain results of known effects. Disease as pertains to the general system is often inscrutable, and medicines only prophetic and problematic in their results. The means for the ends, being always in doubt, until recovery, and then it remains forever a mystery whether the applied medicine, or nature's curative powers peformed the cure. But with us we have a positive art of great usefulness, as it actually corrects disease or restores its ravages, according to the ability of the practitioner engaged. If skillful, he executes what he undertakes to perform, if otherwise, he increases only the demand of skill from some better dentist. For we are never permitted to fail, or to the polished man, it never occurs, at least in the minds of his patients; therefore we would counsel the extreme cultivation of those qualities which beget a confidence completely reliant.

This may be abused, as all virtues are, but it enables the competent person to work in usefulness, and with proper reward, thus bestowing relief for the little ills of life, which when aggregated go far to destroy the comfort and happiness of existence.

I do not presume to lay down a code of laws governing or regulating personal conduct, for fear some "Novice" whom nobody knows forsooth, may attempt to impugn my motives, or fancy results in the hands of gentlemen, only engaged as such, for his use to illustrate quackery. But as all things look yellow to the jaundiced eye, we must exercise compassion for such, and above all avoid even the likelihood of arousing that silly passion, envy.

We believe that every man is capable of receiving instruction from others, even though the source, at times, may be humble and modest. For this reason it is becoming to all to receive with modesty those from whom we certainly can learn something, and we should ever be ready to do justice and honor to the source, however trivial, as a duty to ourselves. The great Sir Walter Scott declared that he owed all his success in life to the invariable habit of learning something from every person he met. Thus, through great personal politeness, he not only received the most prominent success in life, but added to his own comfort as well as to that of others.

With such facts it is hardly necessary to intimate to our younger members, the very great value and necessity of a natural, easy, and generous politeness. Nor is this altogether an inapplicable suggestion to some older members; for the writer has known friends who in visiting towns and villages, calling upon the practising dentists, were so rudely received as to amount to a positive insult. Now we can not afford to have the conduct of an Abernethy without his attainments, at least, and as most make pretensions to a high order of skill, it is the grossest ill-breeding for a man to constitute himself the *Emperor* by this sort of *Fillibustering.* Indeed he must, however patronized, deserve to preside only over the *Ugly Plugs*, with which he no doubt has familiar companionship and daily intercourse. Dentists often run into the error of seeming to be what they are not. Like most other professional men, they imagine the appearances of things will generally answer for the reality. A lawyer or physician may find it sufficient to possess the outward or personal charm of refinement, whilst the dentist must not only be cultivated individually, but have such things around him in his office as bespeak the constant association with liberal and polished people, his rooms decorated, as far as possible with the comforts and elegancies of life.

This is urged not with a view of thus endorsing great ability or skill, but simply as an inducement always effective in drawing the public, to receive the advantages of these qualities, bearing in mind that although such things are no positive evidence of skill, neither are they evidence to the contrary. They merely offer an attractive proof of the gentleman.

Many a patient would as soon enter a dungeon as some dental offices a second time, or come in contact with a ghost as some operators. The austerity of some men, reminds you of Ben Johnson's chagrin with Lord C. without his Tattler, his Shakespeare or his Dictionary to conciliate the vapidity of his acquaintance. Looking back the past year, a beautiful day in June found the writer of this scrap sauntering through a pretty city and noticing the name of a dentist on a door, he concluded to call and endeavor to form the acquaintance of this person. After a *long time* the bell was answered by the robust individual himself, attired as a valet de chamber, according to our ideas. We offered in extenuation of our intrusion, the desire to form his acquaintance, from having frequently heard of him through patients and others and being an entire stranger in the place, we confessed feeling the absence of home and its many kind associations. This could not reconcile the person apparently; for assuming the imperative tone of the Czar, and greatly increasing his own rotundity, he commenced to inform me of the immense importance of his time, etc., but unfortunately being unused to converse with such persons upon door steps, I felt the propriety of an early withdrawal without accomplishing an acquaintance with incivility. Although I have seen some savage races and visited the American wigwam, yet I could not submit to this dentist, nor do I intend that my friends shall ever be exposed to this kind of hospitality. In the KORAN it is related that the Angel took Mahomed on a visiting tour through the seven heavens, paradise and hell, and gave him 90,000 conferences with the Supreme Being, and that, too, within the lapse of an instant. Monstrous as this proposition may seem to the reader, yet the performances of some dentists would hold a respectable position in comparison to this delightful jaunt with the Trumpeter and the long-beard of Mahomedan worship. A Philadelphia friend will (in *silence*) agree with this opinion, if not let him remember the frightful adherence of an exquisite plate, not forgetting the circumstances of its description.

Indeed you are led to believe at times, that to some of our profession, difficulties never occur, failure to reach perfection, a period long since forgotten in the dim past of youth, the accomplishment of wonders, daily occurences—and patients too often impressed with the idea that there is but *one* good dentist in America. A.

